# *r*-2,*c*-6-Bis(2-methoxy­phen­yl)-*t*-3,*t*-5-dimethyl­piperidin-4-one acetic acid solvate

**DOI:** 10.1107/S1600536810017721

**Published:** 2010-05-22

**Authors:** G. Aridoss, S. Sundaramoorthy, D. Velmurugan, K. S. Park, Y. T. Jeong

**Affiliations:** a Department of Image Science and Engineering, Pukyong National University, Busan 608-739, Republic of Korea; b Centre of Advanced Study in Crystallography and Biophysics, University of Madras, Guindy Campus, Chennai 600 025, India

## Abstract

In the title compound, C_21_H_25_NO_3_·C_2_H_4_O_2_, the piperidone ring adopts a chair conformation. The two meth­oxy groups are nearly coplanar with the aromatic rings to which they are attached. The dihedral angle between the two aromatic rings is 60.9 (2)°. There are two short intra­molecular N—H⋯O contacts. The crystal packing is stabilized by inter­molecular O—H⋯N and C—H⋯O inter­actions.

## Related literature

For related structures see: Aridoss *et al.* (2008 ▶),(2009 ▶); Gayathri *et al.* (2008 ▶). For the synthesis of the title compound, see Noller & Baliah (1948 ▶). For ring conformational analysis, see: Cremer & Pople (1975 ▶); Nardelli (1983 ▶).
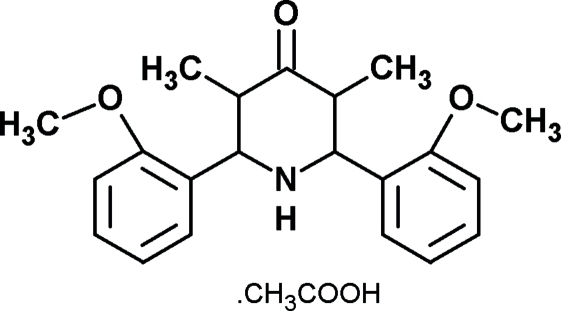


## Experimental

### 

#### Crystal data

C_21_H_25_NO_3_·C_2_H_4_O_2_
*M* *_r_* = 399.47
Triclinic, 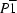

*a* = 9.3059 (5) Å
*b* = 10.7052 (8) Å
*c* = 11.8950 (7) Å
α = 94.432 (3)°
β = 93.341 (2)°
γ = 109.502 (3)°
*V* = 1109.21 (12) Å^3^
*Z* = 2
Mo *K*α radiation
μ = 0.08 mm^−1^
*T* = 292 K
0.25 × 0.23 × 0.2 mm

#### Data collection

Bruker SMART APEXII area-detector diffractometer
Absorption correction: multi-scan (*SADABS*; Bruker, 2008 ▶) *T*_min_ = 0.979, *T*_max_ = 0.983
19986 measured reflections
5528 independent reflections
3271 reflections with *I* > 2σ(*I*)
*R*_int_ = 0.031

#### Refinement

*R*[*F* ^2^ > 2σ(*F* ^2^)] = 0.049
*wR*(*F* ^2^) = 0.173
*S* = 1.00
5528 reflections
271 parameters
H atoms treated by a mixture of independent and constrained refinement
Δρ_max_ = 0.32 e Å^−3^
Δρ_min_ = −0.26 e Å^−3^

### 

Data collection: *APEX2* (Bruker, 2008 ▶); cell refinement: *SAINT* (Bruker, 2008 ▶); data reduction: *SAINT*; program(s) used to solve structure: *SHELXS97* (Sheldrick, 2008 ▶); program(s) used to refine structure: *SHELXL97* (Sheldrick, 2008 ▶); molecular graphics: *ORTEP-3* (Farrugia, 1997 ▶); software used to prepare material for publication: *SHELXL97* and *PLATON* (Spek, 2009 ▶).

## Supplementary Material

Crystal structure: contains datablocks global, I. DOI: 10.1107/S1600536810017721/bt5268sup1.cif

Structure factors: contains datablocks I. DOI: 10.1107/S1600536810017721/bt5268Isup2.hkl

Additional supplementary materials:  crystallographic information; 3D view; checkCIF report

## Figures and Tables

**Table 1:** Hydrogen-bond geometry (Å, °)

*D*—H⋯*A*	*D*—H	H⋯*A*	*D*⋯*A*	*D*—H⋯*A*
O5—H5*A*⋯N1^i^	0.82	1.82	2.642 (2)	178
N1—H1⋯O2	0.869 (18)	2.210 (16)	2.835 (2)	128.6 (14)
N1—H1⋯O3	0.869 (17)	2.322 (18)	2.9241 (17)	126.6 (14)
C19—H19⋯O4^ii^	0.93	2.51	3.441 (2)	175
C22—H22*C*⋯O5^iii^	0.96	2.54	3.482 (3)	167

Symmetry codes: (i) 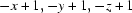; (ii) 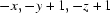; (iii) 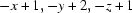.

**Fig. 1.**
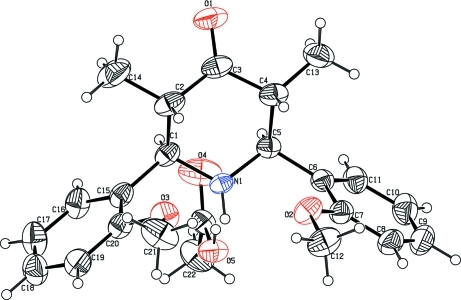
Perspective view of the molecule showing the anisotropic displacement ellipsoids at 30% probability level.

**Fig. 2.**
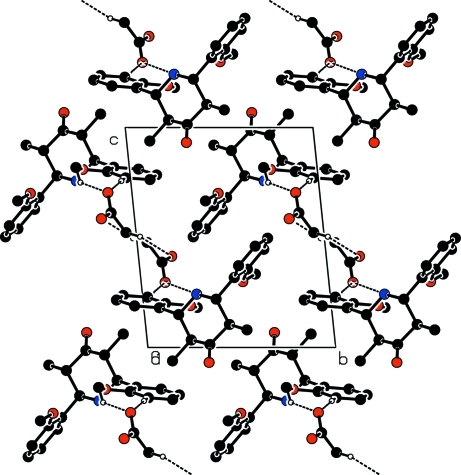
The crystal packing of the molecules viewed down a–axis. For clarity, hydrogen atoms which are not involved in hydrogen bonding are omitted

## supplementary crystallographic information

### Comment

In continuation of our work on establishing the crystal structure and conformation of 2,6-diaryl piperidine-4-ones and their derivatives (Aridoss *et al.*, 2008, 2009 and Gayathri *et al.*, 2008), we are reporting here the crystal structure of the title compound wherein the piperidone ring adopts chair conformation irrespective of the substituents' on both sides of carbonyl and secondary nitrogen in the ring.

In the present structure, the piperidone ring adopts a chair conformation with atoms N1 and C3 deviating by -0.584 (2) and 0.628 (6) Å, respectively,from the least-sqaures plane defined by the remaining atoms (C1/C2/C4/C5) in the ring. When compared with the reported structures of piperidone derivatives (Gayathri *et al.*, 2008), it is clear that the conformation of the piperidone ring is highly influenced by the substitutions at various positions. The molecule is stabilized by N—H···O intramolecular interaction wherein, N1 atom act as a donor to O2 and O3, generating two S(6) motifs. The crystal packing is stabilized by N—H···O, O—H···N and C—H···O intra and intermolecular interactions. The sum of the bond angles around the atom N1(336.6 (3)°) of the piperidone ring in the molecule is in accordance with sp^3^ hybridization.

The puckering parameters (Cremer & Pople, 1975) and the smallest displacement asymmetry parameters (Nardelli, 1983) for the piperidine ring are q_2_ = 0.041 (1) Å, q_3_ = 0.534 (4) Å; Q_T_ = 0.536 (1) Å and θ = 4.40 (1)°,φ_2_ = 135.887 (8)°, respectively.

### Experimental

The title compound was prepared by the condensation of 3-pentanone, 2-methoxybenzaldehyde and ammonium acetate in 1:2:1 molar ratio in ethanol as reported by Noller and Baliah (1948) with slight modification. Diffraction quality white crystal was obtained by recrystalization of the crude sample from ethanol.

### Refinement

H atoms bonded to C and O were positioned geometrically (C—H=0.93-0.98Å, O-H =0.82Å) and allowed to ride on their parent atoms, with 1.5U_eq_(C_methyl_,O) or 1.2 U_eq_(C). The H atom bonded to N was isotropically refined.

### Crystal data

**Table:**

C_21_H_25_NO_3_·C_2_H_4_O_2_	*Z* = 2
*M**_r_* = 399.47	*F*(000) = 428
Triclinic, *P*1	*D*_x_ = 1.196 Mg m^−^^3^
Hall symbol: -P 1	Mo *K*α radiation, λ = 0.71073 Å
*a* = 9.3059 (5) Å	Cell parameters from 3180 reflections
*b* = 10.7052 (8) Å	θ = 1.7–28.4°
*c* = 11.8950 (7) Å	µ = 0.08 mm^−^^1^
α = 94.432 (3)°	*T* = 292 K
β = 93.341 (2)°	Block, colorless
γ = 109.502 (3)°	0.25 × 0.23 × 0.2 mm
*V* = 1109.21 (12) Å^3^	

### Data collection

**Table:**

Bruker SMART APEXII area-detector diffractometer	5528 independent reflections
Radiation source: fine-focus sealed tube	3271 reflections with *I* > 2σ(*I*)
graphite	*R*_int_ = 0.031
ω and φ scans	θ_max_ = 28.4°, θ_min_ = 1.7°
Absorption correction: multi-scan (*SADABS*; Bruker, 2008)	*h* = −12→12
*T*_min_ = 0.979, *T*_max_ = 0.983	*k* = −14→14
19986 measured reflections	*l* = −15→15

### Refinement

**Table:**

Refinement on *F*^2^	Primary atom site location: structure-invariant direct methods
Least-squares matrix: full	Secondary atom site location: difference Fourier map
*R*[*F*^2^ > 2σ(*F*^2^)] = 0.049	Hydrogen site location: inferred from neighbouring sites
*wR*(*F*^2^) = 0.173	H atoms treated by a mixture of independent and constrained refinement
*S* = 1.00	*w* = 1/[σ^2^(*F*_o_^2^) + (0.0885*P*)^2^ + 0.1246*P*] where *P* = (*F*_o_^2^ + 2*F*_c_^2^)/3
5528 reflections	(Δ/σ)_max_ = 0.005
271 parameters	Δρ_max_ = 0.32 e Å^−^^3^
0 restraints	Δρ_min_ = −0.26 e Å^−^^3^

### Special details

**Table:**

Geometry. All esds (except the esd in the dihedral angle between two l.s. planes) are estimated using the full covariance matrix. The cell esds are taken into account individually in the estimation of esds in distances, angles and torsion angles; correlations between esds in cell parameters are only used when they are defined by crystal symmetry. An approximate (isotropic) treatment of cell esds is used for estimating esds involving l.s. planes.
Refinement. Refinement of *F*^2^ against ALL reflections. The weighted *R*-factor wR and goodness of fit *S* are based on *F*^2^, conventional *R*-factors *R* are based on *F*, with *F* set to zero for negative *F*^2^. The threshold expression of *F*^2^ > σ(*F*^2^) is used only for calculating *R*-factors(gt) etc. and is not relevant to the choice of reflections for refinement. *R*-factors based on *F*^2^ are statistically about twice as large as those based on *F*, and *R*- factors based on ALL data will be even larger.

### Fractional atomic coordinates and isotropic or equivalent isotropic displacement parameters (Å^2^)

**Table:**

	*x*	*y*	*z*	*U*_iso_*/*U*_eq_	
O4	0.2740 (2)	0.8721 (2)	0.70402 (14)	0.1087 (6)	
C1	0.43491 (17)	0.18195 (15)	0.14948 (13)	0.0541 (4)	
H1A	0.5080	0.1344	0.1425	0.065*	
C2	0.4178 (2)	0.2350 (2)	0.03418 (14)	0.0678 (5)	
H2	0.3463	0.2842	0.0403	0.081*	
C3	0.5705 (2)	0.33189 (19)	0.01370 (14)	0.0650 (5)	
C4	0.6393 (2)	0.44701 (17)	0.10477 (14)	0.0623 (4)	
H4	0.5714	0.4999	0.1081	0.075*	
C5	0.64521 (17)	0.38984 (14)	0.21975 (12)	0.0505 (4)	
H5	0.7209	0.3445	0.2168	0.061*	
C6	0.70122 (19)	0.49821 (14)	0.31787 (13)	0.0533 (4)	
C7	0.6116 (2)	0.57269 (15)	0.35615 (14)	0.0589 (4)	
C8	0.6675 (3)	0.67106 (17)	0.44625 (16)	0.0746 (5)	
H8	0.6069	0.7190	0.4722	0.089*	
C9	0.8122 (3)	0.6977 (2)	0.49702 (18)	0.0866 (6)	
H9	0.8495	0.7645	0.5569	0.104*	
C10	0.9024 (3)	0.6271 (2)	0.46067 (18)	0.0828 (6)	
H10	1.0005	0.6458	0.4953	0.099*	
C11	0.8457 (2)	0.52775 (17)	0.37193 (16)	0.0659 (5)	
H11	0.9067	0.4794	0.3479	0.079*	
C12	0.3732 (3)	0.6129 (2)	0.33376 (19)	0.0891 (6)	
H12A	0.3540	0.6013	0.4114	0.134*	
H12B	0.2781	0.5795	0.2870	0.134*	
H12C	0.4220	0.7058	0.3261	0.134*	
C13	0.7967 (3)	0.5374 (2)	0.08009 (19)	0.0865 (6)	
H13A	0.8662	0.4883	0.0799	0.130*	
H13B	0.8335	0.6117	0.1374	0.130*	
H13C	0.7897	0.5692	0.0074	0.130*	
C14	0.3526 (3)	0.1237 (3)	−0.06128 (18)	0.1039 (8)	
H14A	0.3557	0.1612	−0.1322	0.156*	
H14B	0.2486	0.0737	−0.0501	0.156*	
H14C	0.4124	0.0659	−0.0619	0.156*	
C15	0.28810 (18)	0.08293 (16)	0.18040 (14)	0.0569 (4)	
C16	0.2765 (2)	−0.04794 (18)	0.18907 (17)	0.0723 (5)	
H16	0.3608	−0.0737	0.1765	0.087*	
C17	0.1441 (2)	−0.1413 (2)	0.2158 (2)	0.0871 (6)	
H17	0.1385	−0.2292	0.2196	0.104*	
C18	0.0207 (2)	−0.1035 (2)	0.2366 (2)	0.0838 (6)	
H18	−0.0684	−0.1659	0.2560	0.101*	
C19	0.0267 (2)	0.0254 (2)	0.22903 (16)	0.0717 (5)	
H19	−0.0577	0.0502	0.2433	0.086*	
C20	0.15927 (18)	0.11803 (17)	0.20012 (14)	0.0600 (4)	
C21	0.0428 (3)	0.2846 (3)	0.1817 (2)	0.0973 (7)	
H21A	−0.0251	0.2319	0.1186	0.146*	
H21B	0.0704	0.3773	0.1706	0.146*	
H21C	−0.0075	0.2686	0.2500	0.146*	
N1	0.49978 (14)	0.28941 (12)	0.24234 (10)	0.0474 (3)	
O1	0.63714 (19)	0.31782 (17)	−0.06797 (12)	0.0943 (5)	
O2	0.47078 (15)	0.54141 (12)	0.29933 (11)	0.0716 (4)	
O3	0.17552 (13)	0.24937 (12)	0.19021 (12)	0.0731 (4)	
H1	0.4328 (19)	0.3276 (16)	0.2563 (14)	0.057 (4)*	
C22	0.2703 (3)	0.9465 (3)	0.5224 (2)	0.0980 (7)	
H22A	0.1935	0.9802	0.5490	0.147*	
H22B	0.2283	0.8846	0.4562	0.147*	
H22C	0.3560	1.0191	0.5037	0.147*	
C23	0.32168 (18)	0.87804 (16)	0.61225 (16)	0.0609 (4)	
O5	0.42181 (15)	0.82324 (12)	0.58533 (10)	0.0707 (4)	
H5A	0.4448	0.7887	0.6396	0.106*	

### Atomic displacement parameters (Å^2^)

**Table:**

	*U*^11^	*U*^22^	*U*^33^	*U*^12^	*U*^13^	*U*^23^
O4	0.1096 (12)	0.1637 (16)	0.1017 (12)	0.0972 (12)	0.0448 (10)	0.0488 (11)
C1	0.0527 (9)	0.0637 (9)	0.0533 (9)	0.0328 (7)	0.0022 (7)	−0.0067 (7)
C2	0.0644 (11)	0.0993 (13)	0.0521 (10)	0.0474 (10)	0.0012 (8)	−0.0032 (9)
C3	0.0784 (12)	0.0886 (12)	0.0474 (9)	0.0524 (10)	0.0108 (8)	0.0105 (8)
C4	0.0791 (11)	0.0689 (10)	0.0558 (9)	0.0436 (9)	0.0180 (8)	0.0150 (8)
C5	0.0546 (9)	0.0532 (8)	0.0522 (9)	0.0279 (7)	0.0105 (7)	0.0074 (6)
C6	0.0645 (10)	0.0476 (8)	0.0509 (9)	0.0214 (7)	0.0101 (7)	0.0100 (6)
C7	0.0749 (11)	0.0507 (8)	0.0555 (9)	0.0261 (8)	0.0117 (8)	0.0064 (7)
C8	0.1046 (16)	0.0570 (10)	0.0654 (11)	0.0316 (10)	0.0157 (11)	0.0011 (8)
C9	0.1166 (18)	0.0630 (11)	0.0681 (12)	0.0196 (12)	−0.0063 (12)	−0.0067 (9)
C10	0.0855 (14)	0.0684 (12)	0.0818 (13)	0.0133 (10)	−0.0152 (11)	0.0076 (10)
C11	0.0691 (11)	0.0575 (9)	0.0707 (11)	0.0205 (8)	0.0032 (9)	0.0107 (8)
C12	0.1012 (16)	0.0992 (15)	0.0907 (15)	0.0668 (13)	0.0186 (12)	−0.0036 (11)
C13	0.1080 (17)	0.0800 (13)	0.0780 (13)	0.0317 (12)	0.0350 (12)	0.0286 (10)
C14	0.0884 (15)	0.150 (2)	0.0606 (13)	0.0355 (15)	−0.0106 (11)	−0.0285 (13)
C15	0.0508 (9)	0.0637 (9)	0.0575 (9)	0.0255 (7)	−0.0010 (7)	−0.0103 (7)
C16	0.0595 (11)	0.0656 (11)	0.0921 (14)	0.0272 (9)	−0.0021 (9)	−0.0101 (9)
C17	0.0714 (13)	0.0614 (11)	0.1236 (19)	0.0202 (10)	−0.0025 (12)	−0.0002 (11)
C18	0.0596 (11)	0.0755 (13)	0.1040 (16)	0.0105 (9)	0.0009 (10)	−0.0023 (11)
C19	0.0513 (10)	0.0833 (13)	0.0797 (13)	0.0254 (9)	0.0025 (8)	−0.0048 (10)
C20	0.0529 (9)	0.0651 (10)	0.0644 (10)	0.0265 (8)	0.0010 (7)	−0.0066 (8)
C21	0.0762 (13)	0.1153 (17)	0.131 (2)	0.0613 (13)	0.0365 (13)	0.0465 (15)
N1	0.0480 (7)	0.0521 (7)	0.0486 (7)	0.0264 (6)	0.0079 (5)	0.0001 (5)
O1	0.1128 (11)	0.1183 (12)	0.0627 (8)	0.0504 (9)	0.0354 (8)	0.0029 (7)
O2	0.0794 (8)	0.0754 (8)	0.0738 (8)	0.0476 (7)	0.0080 (7)	−0.0074 (6)
O3	0.0597 (7)	0.0747 (8)	0.0979 (10)	0.0392 (6)	0.0169 (6)	0.0049 (7)
C22	0.0881 (15)	0.1015 (16)	0.1127 (18)	0.0350 (12)	0.0098 (13)	0.0478 (14)
C23	0.0485 (9)	0.0621 (10)	0.0718 (11)	0.0157 (7)	0.0113 (8)	0.0145 (8)
O5	0.0856 (9)	0.0786 (8)	0.0611 (7)	0.0406 (7)	0.0226 (6)	0.0175 (6)

### Geometric parameters (Å, °)

**Table:**

O4—C23	1.200 (2)	C12—H12C	0.9600
C1—N1	1.4731 (19)	C13—H13A	0.9600
C1—C15	1.509 (2)	C13—H13B	0.9600
C1—C2	1.544 (2)	C13—H13C	0.9600
C1—H1A	0.9800	C14—H14A	0.9600
C2—C3	1.501 (3)	C14—H14B	0.9600
C2—C14	1.518 (3)	C14—H14C	0.9600
C2—H2	0.9800	C15—C16	1.381 (2)
C3—O1	1.208 (2)	C15—C20	1.398 (2)
C3—C4	1.513 (3)	C16—C17	1.375 (3)
C4—C13	1.522 (3)	C16—H16	0.9300
C4—C5	1.546 (2)	C17—C18	1.369 (3)
C4—H4	0.9800	C17—H17	0.9300
C5—N1	1.4748 (19)	C18—C19	1.372 (3)
C5—C6	1.515 (2)	C18—H18	0.9300
C5—H5	0.9800	C19—C20	1.381 (3)
C6—C11	1.380 (2)	C19—H19	0.9300
C6—C7	1.404 (2)	C20—O3	1.378 (2)
C7—O2	1.362 (2)	C21—O3	1.407 (2)
C7—C8	1.387 (3)	C21—H21A	0.9600
C8—C9	1.372 (3)	C21—H21B	0.9600
C8—H8	0.9300	C21—H21C	0.9600
C9—C10	1.371 (3)	N1—H1	0.869 (17)
C9—H9	0.9300	C22—C23	1.485 (3)
C10—C11	1.382 (3)	C22—H22A	0.9600
C10—H10	0.9300	C22—H22B	0.9600
C11—H11	0.9300	C22—H22C	0.9600
C12—O2	1.427 (2)	C23—O5	1.298 (2)
C12—H12A	0.9600	O5—H5A	0.8200
C12—H12B	0.9600		
			
N1—C1—C15	110.18 (11)	C4—C13—H13A	109.5
N1—C1—C2	112.74 (13)	C4—C13—H13B	109.5
C15—C1—C2	113.26 (13)	H13A—C13—H13B	109.5
N1—C1—H1A	106.7	C4—C13—H13C	109.5
C15—C1—H1A	106.7	H13A—C13—H13C	109.5
C2—C1—H1A	106.7	H13B—C13—H13C	109.5
C3—C2—C14	113.04 (15)	C2—C14—H14A	109.5
C3—C2—C1	107.96 (13)	C2—C14—H14B	109.5
C14—C2—C1	112.43 (18)	H14A—C14—H14B	109.5
C3—C2—H2	107.7	C2—C14—H14C	109.5
C14—C2—H2	107.7	H14A—C14—H14C	109.5
C1—C2—H2	107.7	H14B—C14—H14C	109.5
O1—C3—C2	122.82 (18)	C16—C15—C20	117.43 (16)
O1—C3—C4	121.76 (18)	C16—C15—C1	120.30 (14)
C2—C3—C4	115.39 (14)	C20—C15—C1	122.27 (15)
C3—C4—C13	112.14 (15)	C17—C16—C15	121.94 (17)
C3—C4—C5	108.37 (13)	C17—C16—H16	119.0
C13—C4—C5	111.53 (16)	C15—C16—H16	119.0
C3—C4—H4	108.2	C18—C17—C16	119.30 (19)
C13—C4—H4	108.2	C18—C17—H17	120.4
C5—C4—H4	108.2	C16—C17—H17	120.4
N1—C5—C6	110.18 (11)	C17—C18—C19	120.87 (19)
N1—C5—C4	114.00 (13)	C17—C18—H18	119.6
C6—C5—C4	112.25 (12)	C19—C18—H18	119.6
N1—C5—H5	106.6	C18—C19—C20	119.44 (17)
C6—C5—H5	106.6	C18—C19—H19	120.3
C4—C5—H5	106.6	C20—C19—H19	120.3
C11—C6—C7	117.73 (15)	O3—C20—C19	123.47 (15)
C11—C6—C5	119.99 (14)	O3—C20—C15	115.54 (15)
C7—C6—C5	122.28 (15)	C19—C20—C15	120.99 (16)
O2—C7—C8	123.97 (16)	O3—C21—H21A	109.5
O2—C7—C6	115.70 (14)	O3—C21—H21B	109.5
C8—C7—C6	120.33 (18)	H21A—C21—H21B	109.5
C9—C8—C7	119.97 (19)	O3—C21—H21C	109.5
C9—C8—H8	120.0	H21A—C21—H21C	109.5
C7—C8—H8	120.0	H21B—C21—H21C	109.5
C8—C9—C10	120.81 (18)	C1—N1—C5	113.75 (11)
C8—C9—H9	119.6	C1—N1—H1	109.1 (11)
C10—C9—H9	119.6	C5—N1—H1	110.0 (11)
C9—C10—C11	119.2 (2)	C7—O2—C12	118.78 (15)
C9—C10—H10	120.4	C20—O3—C21	118.41 (15)
C11—C10—H10	120.4	C23—C22—H22A	109.5
C6—C11—C10	121.98 (18)	C23—C22—H22B	109.5
C6—C11—H11	119.0	H22A—C22—H22B	109.5
C10—C11—H11	119.0	C23—C22—H22C	109.5
O2—C12—H12A	109.5	H22A—C22—H22C	109.5
O2—C12—H12B	109.5	H22B—C22—H22C	109.5
H12A—C12—H12B	109.5	O4—C23—O5	121.44 (16)
O2—C12—H12C	109.5	O4—C23—C22	122.88 (18)
H12A—C12—H12C	109.5	O5—C23—C22	115.69 (17)
H12B—C12—H12C	109.5	C23—O5—H5A	109.5
			
N1—C1—C2—C3	53.96 (16)	C8—C9—C10—C11	−0.2 (3)
C15—C1—C2—C3	179.92 (12)	C7—C6—C11—C10	−0.2 (3)
N1—C1—C2—C14	179.33 (14)	C5—C6—C11—C10	179.02 (16)
C15—C1—C2—C14	−54.70 (18)	C9—C10—C11—C6	0.7 (3)
C14—C2—C3—O1	−3.6 (3)	N1—C1—C15—C16	−117.88 (16)
C1—C2—C3—O1	121.43 (18)	C2—C1—C15—C16	114.79 (18)
C14—C2—C3—C4	178.51 (16)	N1—C1—C15—C20	62.78 (19)
C1—C2—C3—C4	−56.48 (18)	C2—C1—C15—C20	−64.54 (19)
O1—C3—C4—C13	−0.3 (2)	C20—C15—C16—C17	0.1 (3)
C2—C3—C4—C13	177.66 (15)	C1—C15—C16—C17	−179.23 (18)
O1—C3—C4—C5	−123.82 (18)	C15—C16—C17—C18	−1.3 (3)
C2—C3—C4—C5	54.13 (19)	C16—C17—C18—C19	1.2 (3)
C3—C4—C5—N1	−49.38 (17)	C17—C18—C19—C20	0.0 (3)
C13—C4—C5—N1	−173.28 (13)	C18—C19—C20—O3	179.78 (18)
C3—C4—C5—C6	−175.55 (13)	C18—C19—C20—C15	−1.1 (3)
C13—C4—C5—C6	60.54 (18)	C16—C15—C20—O3	−179.77 (15)
N1—C5—C6—C11	124.36 (15)	C1—C15—C20—O3	−0.4 (2)
C4—C5—C6—C11	−107.43 (17)	C16—C15—C20—C19	1.1 (3)
N1—C5—C6—C7	−56.50 (18)	C1—C15—C20—C19	−179.58 (16)
C4—C5—C6—C7	71.71 (18)	C15—C1—N1—C5	179.12 (12)
C11—C6—C7—O2	179.10 (14)	C2—C1—N1—C5	−53.27 (16)
C5—C6—C7—O2	−0.1 (2)	C6—C5—N1—C1	178.43 (12)
C11—C6—C7—C8	−0.8 (2)	C4—C5—N1—C1	51.18 (16)
C5—C6—C7—C8	−179.95 (15)	C8—C7—O2—C12	−0.2 (3)
O2—C7—C8—C9	−178.64 (17)	C6—C7—O2—C12	179.87 (16)
C6—C7—C8—C9	1.2 (3)	C19—C20—O3—C21	−16.0 (3)
C7—C8—C9—C10	−0.7 (3)	C15—C20—O3—C21	164.90 (17)

### Hydrogen-bond geometry (Å, °)

**Table:**

*D*—H···*A*	*D*—H	H···*A*	*D*···*A*	*D*—H···*A*
O5—H5A···N1^i^	0.82	1.82	2.642 (2)	178
N1—H1···O2	0.869 (18)	2.210 (16)	2.835 (2)	128.6 (14)
N1—H1···O3	0.869 (17)	2.322 (18)	2.9241 (17)	126.6 (14)
C19—H19···O4^ii^	0.93	2.51	3.441 (2)	175
C22—H22C···O5^iii^	0.96	2.54	3.482 (3)	167

Symmetry codes: (i) −*x*+1, −*y*+1, −*z*+1; (ii) −*x*, −*y*+1, −*z*+1; (iii) −*x*+1, −*y*+2, −*z*+1.
